# Monitoring the Effectiveness of High-Fluence Peripheral Crosslinking for Corneal Neovascularization with Anterior Segment Optical Coherence Tomography Angiography

**DOI:** 10.3390/jcm13133804

**Published:** 2024-06-28

**Authors:** Ruth Donner, Gerald Schmidinger, Michal Klimek, Julia Aschauer

**Affiliations:** Department of Ophthalmology and Optometry, Medical University of Vienna, Spitalgasse 23, 1090 Vienna, Austria; gerald.schmidinger@meduniwien.ac.at (G.S.); n12117073@students.meduniwien.ac.at (M.K.); julia.aschauer@meduniwien.ac.at (J.A.)

**Keywords:** angioregression, risk management

## Abstract

**Background/Aims:** To investigate the effectiveness of an accelerated high-fluence peripheral crosslinking (pCXL) treatment protocol for corneal neovascularization (cNV) and the viability of optical coherence tomography angiography (OCTA) to monitor cNV dynamics. **Methods:** This pilot study included six eyes of six adult patients with cNV in at least one corneal quadrant who were treated with pCXL (7.2 J/cm^2^, 9 mW). The degree of cNV regression was monitored with slit lamp photography and anterior segment OCTA. The main outcome measure was total vessel area one and four weeks after treatment. **Results:** OCTA allowed for the objective monitoring of vascular metrics: The total vessel area declined from an average of 1025.4 mm^2^ (min: 0.13 mm^2^; max: 3637 mm^2^) at the baseline evaluation to 382.4 mm^2^ (min: 0.08 mm^2^; max: 1528 mm^2^) (*p* = 0.096). The total vessel length lessened from an average of 107.1 mm (min: 2.8 mm; max: 321.1 mm) to 47 mm (min: 2.6 mm; max: 156.5 mm) (*p*= 0.27). The average number of junctions at baseline decreased from 46.67 (min: 3; max: 166) to 26.5 (min: 0; max: 50) (*p* = 0.23). The junction density decreased from an average of 10.75/mm^2^ (min: 0.0002 /mm^2^; max: 36.5056/mm^2^) to 7.37/mm^2^ (avg.) (min: 0; max 18.7356/mm^2^) (*p* = 0.24). PCXL was performed safely without adverse effects, but vascular occlusion was not complete in all eyes. **Conclusions:** High-fluence pCXL may represent a valuable treatment option to achieve cNV regression, whilst the optimal fluence dose still remains to be defined. Anterior segment OCTA is an innovative tool for non-invasive, objective, and quantitative cNV monitoring.

## 1. Introduction

Corneal neovascularization (cNV) entails the growth of blood vessels into the physiologically avascular cornea and is provoked by various factors including inflammation, infection, trauma, or underlying systemic diseases [1]. This can interfere with the cornea’s transparency due to light scattering, scarring, and lipid deposition and is a known risk factor for corneal graft rejection.

In an avascular state, the cornea is characterized by its unique immune privilege, which allows for remarkably low rates of rejection after transplantation compared to other organs [2,3]. While the rate of graft survival at 5 years in a low-risk recipient is 90%, high-risk recipients experience survival rates under 50% at one year and ≤10% after two years [4]. Features that contribute to the qualification of a recipient as being at high risk for rejection include a history of graft rejection, significant cNV, and active inflammation [1]. Since cNV signifies a considerable risk for rejection, preconditioning treatment appears to be indicated prior to keratoplasty. However, all possible treatment options (diathermy, argon laser coagulation, Mitomycin-C chemoembolization) may lead to inflammation and an increased risk of rejection in the early postoperative phase. Thus, careful documentation and follow-up is imperative. Currently, no data on the risk of rejection are known for any of these treatment options, and further, there are no available clinical comparisons between the listed treatments.

Various treatments are available to combat cNV, often in a big-picture goal to support corneal graft acceptance and survival. The choice of treatment depends on the underlying cause, severity of the condition, and other factors such as the patient’s age and general health. Among possible treatments are topical or systemic medications, including anti-inflammatory drugs (such as corticosteroids), fine needle diathermy, laser treatment, injection of Mitomycin C (MICE), as well as vascular endothelial growth factor (VEGF) inhibitors including bevacizumab, which can help reduce inflammation and slow the growth of new blood and lymph vessels, which appear to correlate [1,5,6,7,8,9,10,11,12,13,14]. Recently, peripheral corneal crosslinking (pCXL) has been gaining interest in this indication profile due to its ability to induce the apoptosis of vascular endothelial cells [15,16]. Further, a marked advantage to crosslinking is its ability to not only achieve damage to unwanted blood vessels but to simultaneously damage the associated lymph vessels in the treated area [15]. Due to lymph vessels’ significant role in rejection reactions, prohibiting excessive growth is likely to have a beneficial effect on high-risk recipient beds.

Due to the dynamic nature of vessel development and the tendency for an amplifying feedback loop, treatment combating cNV is a demanding task. Currently, there is a lack of quantifiable, high-quality imaging techniques for cNV [17]. While slit lamp photography is a useful aid in general anterior segment imaging, it does not suffice as a sole imaging device for cNV due to its inability to clearly visualize arteries, vessels being masked by opacities (such as scarring), as well as difficulties depicting small capillaries. Fluorescein angiography (FA) and indocyanine green angiography (ICGA) offer a more precise depiction of vessels, but they always represent invasive and resource-consuming procedures, where evaluation is dependent on subjective grading. Optical coherence tomography angiography (OCTA) is an already established technique for retinal imaging, and early applications to the anterior segment show positive results [18,19]. Using its ability to quantify vascular activity in the cornea could signify a considerable improvement in the quality of cNV documentation, which may influence future decision-making regarding treatment protocols essentially.

Therefore, the central objective of this pilot study is twofold: to demonstrate the effectiveness and short-term safety of pCXL with high-fluence settings for the treatment of cNV and to quantitatively analyze any given induction of angioregression with pCXL using OCTA imaging compared to slit lamp photography.

## 2. Methods

This retrospective pilot study was approved by the Ethics Committee of the Medical University of Vienna (EK 1295/2022) and included six patients with significant cNV in at least one corneal quadrant of one affected eye, who’s minimal pachymetry exceeded 350 µm at the cNV location of interest, and who showed no indication of active herpetic or other infectious corneal disease, uncontrolled atopic disease, or uncontrolled glaucoma. Only patients with cNV that were non-responsive to topical steroid therapy were offered pCXL.

Six eyes of six patients (five right eyes, one left eye) were included. Two patients were female and four were male, aged between 49 and 77 (mean 58 ± 9.9 years). Visual acuity prior to treatment was 0.76 logMAR on average (min 1.0 logMAR; max 0.3 logMAR) and showed no change after treatment. The underlying etiology of the cNV included postherpetic neovascularization (*n* = 2) and neovascularization after corneal transplantation (*n* = 4) (Table 1). Patients’ informed consent was obtained before imaging and treatment.

Swept-source OCTA of the cornea (Plex Elite 9000, Carl Zeiss Meditec, Dublin, CA, USA) was performed with a 10-diopter adaptor lens prior to pCXL and as a part of the 1 week and 1 month follow-up. At each session, 3 × 3 mm as well as 6 × 6 mm scan patterns were performed centered on the corneal apex. Segmentation needed to be performed manually on each B scan, delineating the epithelial and endothelial surfaces. The resulting images allowed for an interpretation of the cNV maximal depth (posterior border of the deepest flow signal from epithelial surface, µm) and the cNV depth (=ratio between cNV thickness and total corneal thickness, %) per software available with the OCTA device. FIJI (ImageJ 2.0.0, imagej.net) was then used to delineate the limbus. Any image details beyond this border as well as corneal background flow noise were masked in black to allow for accurate vessel measurements. Further analysis was performed using Angiotool (0.6a, National Cancer Institute, Bethesda, MD, USA), which is able to identify the total area of the vascular complex (CV lesion size, mm^2^) [6,7,8]. This tool is further able to calculate vessel characteristics, including total vessel length (mm), number of vessel junctions, and vessel junction density (n/mm^2^).

Slit lamp color photography (SL 800, Carl Zeiss Meditec, Dublin, CA, USA) was additionally performed at each visit, with a focus on the corneal vascularization/scar/opacity, using 10- and 16-fold magnifications with diffuse illumination. Quality grading was performed for each acquired image. Only images graded to be of a high quality regarding the focus on the region of interest and resolution of the vessel were included in the data analysis as a measure of quality control.

CXL was performed epi-off in the area of the vascular net using a 0.1% riboflavin solution in 20% dextran phosphate sodium (Peschke D, PESCHKE Trade GmbH, Huenenberg, Switzerland) per a well-recognized procedure (one drop every 2 min for 20 min). An accelerated protocol using 9 mW with a total energy dosage of 7.2 J/cm^2^ was performed for all patients with UV-impermeable protection of the limbus and cornea outside of the treated area by use of a custom-made stencil for each eye in place during treatment. Postoperative care followed a standardized procedure including steroid (Prednifluid, Dermapharm GmbH, Vienna, Austria) and antibiotic eye drops (Gentax, AGEPHA Pharma, Senec, Slovakia), as well as bandage lenses. Follow-up visits were planned according to standard clinical procedure at the 1- and 4-week marks, at which imaging was repeated in addition to standard ophthalmic examination.

## 3. Results

None of the patients reported significant pain following pCXL, and there were no adverse events after treatment such as persistent peri- and postoperative bleeding, delayed healing patterns with persistent epithelial defects, or signs of limbal stem cell deficiency.

Qualitatively, the identification and follow-up of vessels were considerably more difficult in images obtained with slit lamp photography rather than the isolated vessels depicted via OCTA.

Quantitative image analysis showed a decline in the total vessel area from an average of 1025.4 mm^2^ (min: 0.13 mm^2^; max: 3637 mm^2^) at the baseline evaluation to 382.4 mm^2^ (min: 0.08 mm^2^; max: 1528 mm^2^) at the one-month follow-up (*p* = 0.096). Total vessel length was reduced from an average of 107.1 mm (min: 2.8 mm; max: 321.1 mm) to 47 mm (min: 2.6 mm; max: 156.5 mm) (*p* = 0.27). The average number of junctions at baseline was 46.67 (min: 3; max: 166) and 26.5 (min: 0; max: 50) after one month (*p* = 0.23), and junction density was reduced from an average of 10.75/mm^2^ (min: 0.0002/mm^2^; max: 36.5056/mm^2^) to an average of 7.37/mm^2^ (min: 0; max 18.7356/mm^2^) (*p* = 0.24).

**Figure 1:**
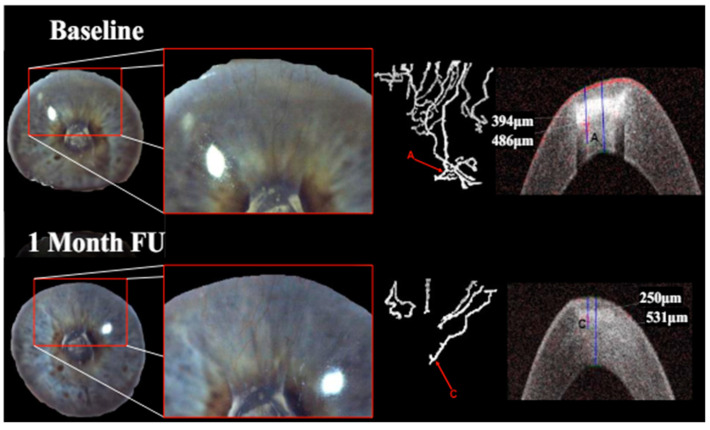
Patient with corneal neovascularization (CNV) at baseline and 1-month follow-up (FU): overview as well as close-up of the region of interest with diffuse illumination in slit lamp photography (far-left image with close-up to its right). Isolation of perfused blood vessels via OCTA imaging (center-right and far-right images). Identification of vessel depth via signal response in OCTA B-scan (vessel A and vessel C). Incomplete angioregression was observed in this patient.

**Figure 2:**
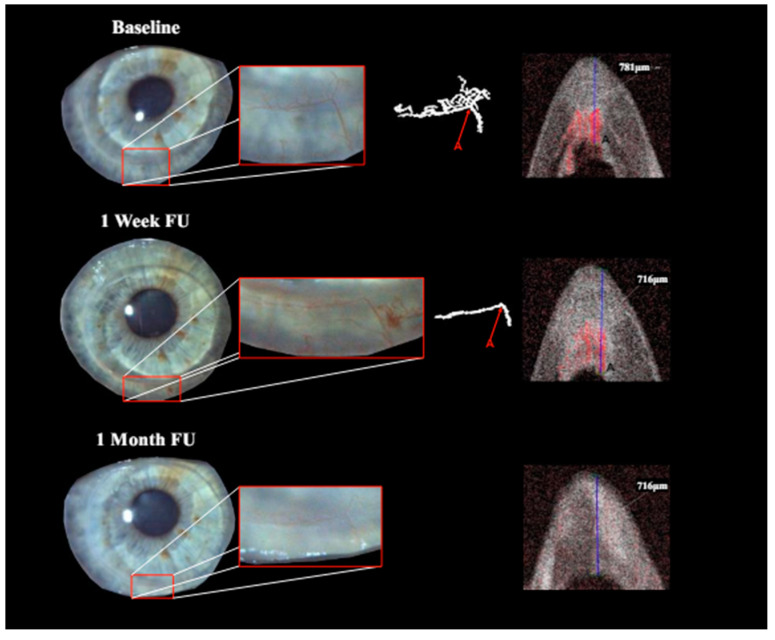
Patient with corneal neovascularization (CNV) at baseline, 1-week and 1-month follow-up (FU): overview and close-up of the region of interest with diffuse illumination in slit lamp photography (far-left image with close-up to its right). Isolation of perfused blood vessels via OCTA imaging (center-right and far-right images). Identification of vessel depth via signal response in OCTA B-scan (vessel A). Uncertain identification of vessel perfusion in slit lamp photography at 1-week FU due to masking by a small area of bleeding in resorption, yet clarity in OCTA imaging (vessel A). Complete regression was achieved by month 1.

**Figure 3:**
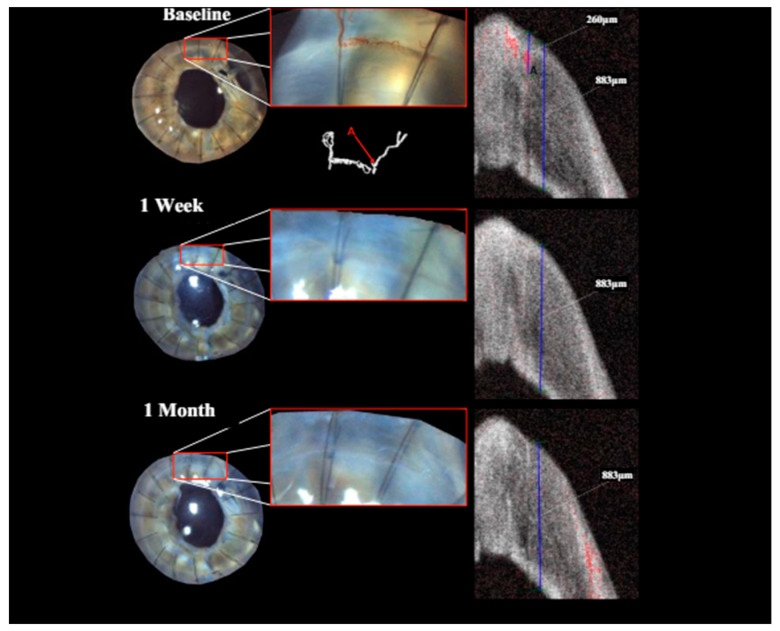
Patient with corneal neovascularization (CNV) at baseline, 1-week and 1-month follow-up (FU): overview and close-up of the region of interest with diffuse illumination in slit lamp photography (far-left image with close-up to its right). Isolation of perfused blood vessels via OCTA imaging (below close-up of baseline slit lamp photography). Complete and persistent angioregression was achieved in this patient as early as by week one.

**Figure 4:**
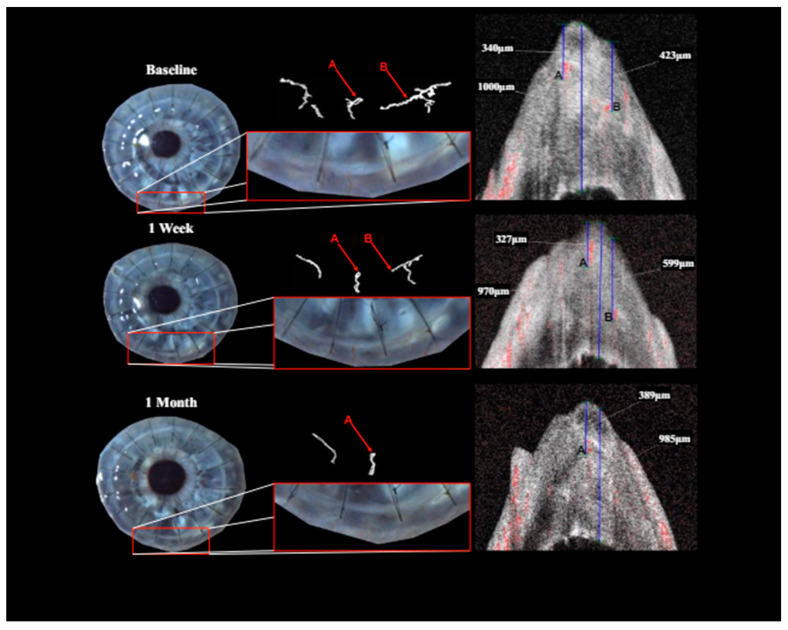
Patient with corneal neovascularization (CNV) at baseline, 1-week and 1-month follow-up (FU): overview as well as close-up of the region of interest with diffuse illumination in slit lamp photography (far-left image with close-up to its right). Isolation of perfused blood vessels via OCTA imaging (uppermost image in the center and far-right image). Identification of vessel depth via signal response in OCTA B-scan (vessel A and vessel B). While vessel B showed complete regression, vessel A remained.

Treatment with pCXL achieved a visible angioregressive effect in the targeted areas in all corneas, as could be observed in processed OCTA images as well as in slit lamp photography. Quantitatively, vessel area was reduced from an average of 31.7% (min 7.7%, max 60.4%) to an average of 21.2% (min 6.7%, max 34.1%) (*p* = 0.069). The level of detail regarding vessel conformity, location, and depth given by OCTA imaging stands out in comparison to photography, as can be seen in Figure 1, Figure 2 and Figure 3. Also, imaging could clearly demonstrate that the effect achievable with the applied protocol led to complete and immediate angioregression in several cases (ass illustrated exemplarily by Figure 3 and Figure 4). In some eyes though, complete angioregression could only be observed with some delay (see Figure 2), whilst in further cases, the observed effect of vessel regression was incomplete until month 1 (see Figure 1).

## 4. Discussion

Prior to high-risk corneal transplantation, optimal preconditioning of the recipient by mitigating risk factors for rejection is a central concern. However, the treatment of cNV is a difficult and complex task. Finding an effective treatment is made even more difficult by suboptimal available documentation possibilities. Verbal description is subject to immense levels of subjectivity and while slit lamp photography offers an objective method of documentation, it lacks the ability for quantitative analysis and does not always allow for visualization of deep stromal cNV, nor does it mark activity. While accurate and objective, fluorescein and indocyanine green angiography techniques are time-consuming and invasive, requiring a somewhat strict indication for repeated and frequent imaging. Thus, imaging via OCTA represents a leap in the quality of documentation for cNV. Further, being able to quantify vasculature is an opportunity for very high degrees of objectivity and comparability.

This retrospective pilot study shows that OCTA imaging and analysis is uniquely capable of providing detailed objective imaging of and quantitative data for monitoring cNV. Seeing as this trial is also the first to apply high-fluence CXL to the peripheral cornea, documenting its effectiveness as well as possible side effects was a major objective. While no adverse effects could be observed, several patients showed incomplete vessel regression of treated vessels within the follow-up period. 

The achieved treatment effect could be shown very well visually as well as in quantitative data. Repeated measurements during the follow-up period showed that this imaging technique is replicable and user-friendly. A welcome benefit to this technique is that a technician can perform imaging independent of a physician’s availability (compared to FLA/ICGA), making it a particularly time-saving tool for standard clinical practice. 

While OCTA images clearly offered a more advanced analysis of vessel dynamics due to the isolation from overshadowing structures and the ability to quantify vessel characteristics compared to slit lamp photography, segmentation and analysis of raw OCTA images to achieve images suited for further interpretation is time- and skill-intensive. Currently, this is the main barrier to its routine clinical use for cNV analysis. With advances in interpretation software for corneal imaging, OCTA may become a viable clinical tool.

With increasing use of artificial intelligence (AI) in clinical ophthalmology, the quality of imaging found with OCTA could allow AI to become a viable and valuable tool for the segmentation and analysis of cNV.

Also, despite promising results, it is imperative to consider the limitations of this trial. The current results are based on a fairly short follow-up period. Further analyses will offer insight into the long-term effectiveness of pCXL for this indication profile. The small number of included patients further makes it unlikely to reach significant statistical results. However, this is the first trial to show that high-fluence pCXL is an effective treatment option for cNV via non-invasive OCTA imaging, and short-term effectiveness is a crucial marker when angioregressive treatment is used as a preconditioning measure prior to keratoplasty. At least for the immediate postoperative phase, this treatment proved safe in this pilot study. These results pave the way for future larger-scale, longitudinal studies to investigate the optimal fluence effective for treating corneal lymph and blood vessels as well as the identification of the most ideal vascular parameters for monitoring the effectiveness of treatment.

Several explanations could account for the insufficient effect that was observed after pCXL in some patients. One possible explanation may be that the CXL effect may not have reached the depth of some of the targeted deeper vessels. The results from an earlier laboratory study suggest that an increase in fluence under consistent intensity (9 mW) could be an appropriate adjustment to reach vessels in the deep stroma [20]. Another hurdle in effective angioregression could be vessel size—large established vessels showing a high level of resistance to treatment. To increase effectiveness, combining pCXL with other treatments such as fine needle diathermy or MICE (to close the lumen of primary large feeder vessels) may be useful. Lastly, in order to avoid reperfusion after an initially satisfactory effect, one may consider following pCXL up with anti-VEGF therapy to combat any VEGF upregulation that may promote reperfusion.

This trial evaluated the effectiveness of high-fluence accelerated pCXL with non-invasive monitoring using innovative anterior segment OCTA. The level of detail in the documentation of cNV, extending to its pro- and regression, that is achievable with OCTA imaging signifies much needed progress for anterior segment imaging. Being able to accurately document cNV allows for a more sophisticated and nuanced evaluation of treatment effect. As became apparent, the applied protocol showed promising angioregression without adverse effects, but it was insufficient in achieving complete angioregression in some cases, indicating the need for further adjustment in the treatment protocol. The ability to perform reliable and non-invasive cNV imaging bears the potential to play an essential role in future development and decisions regarding angioregressive therapy of the cornea.

## Figures and Tables

**Table 1:** Patient characteristics including gender, age, visual acuity, and vascular aetiology.

Patient	Gender	Age	Visual Acuity Prior to Treatment	Visual Acuity after Treatment	Vascular Aetiology
1	Female	52	0.6	0.6	Post-KP
2	Male	49	0.8	0.8	Post-KP
3	Female	60	0.7	0.7	Herpetic
4	Male	56	0.7	0.6	Post-KP
5	Male	77	0.8	0.8	Herpetic
6	Male	62	1	0.9	Post-KP

## Data Availability

The data presented in this study are available on request from the corresponding author due to patient privacy interests.
